# PW06 Triggered Fas-FADD to Induce Apoptotic Cell Death In Human Pancreatic Carcinoma MIA *PaCa-2* Cells through the Activation of the Caspase-Mediated Pathway

**DOI:** 10.1155/2023/3479688

**Published:** 2023-02-11

**Authors:** Yi-Ping Huang, Te-Chun Hsia, Chun-An Yeh, Yi-Shih Ma, Sheng-Yao Hsu, Yi-Chung Liu, Ping-Chiang Lyu, Kuang-Chi Lai, Shu-Fen Peng, Jin-Cherng Lien, Wen-Tsong Hsieh

**Affiliations:** ^1^Department of Physiology, School of Medicine, China Medical University, Taichung 404333, Taiwan; ^2^Department of Medical Research, China Medical University Hospital, Taichung 404333, Taiwan; ^3^Department of Respiratory Therapy, China Medical University, Taichung 404333, Taiwan; ^4^School of Chinese Medicine for Post-Baccalaureate, I-Shou University, Kaohsiung 84001, Taiwan; ^5^Department of Chinese Medicine, E-Da Hospital, Kaohsiung 82385, Taiwan; ^6^Department of Ophthalmology, An Nan Hospital, China Medical University, Tainan 709, Taiwan; ^7^Department of Optometry, Chung Hwa University of Medical Technology, Tainan 717302, Taiwan; ^8^Institute of Population Health Sciences, National Health Research Institutes, Miaoli 350, Taiwan; ^9^Institute of Bioinformatics and Structural Biology, National Tsing-Hua University, Hsinchu 300044, Taiwan; ^10^Department of Medical Laboratory Science and Biotechnology, College of Medical Technology, Chung Hwa University of Medical Technology, Tainan, Taiwan; ^11^Department of Biological Science and Technology, China Medical University, Taichung 406040, Taiwan; ^12^Department of Internal Medicine, China Medical University Hospital, Taichung 404333, Taiwan; ^13^School of Pharmacy, China Medical University, Taichung 406040, Taiwan; ^14^Chinese Medicine Research Center, China Medical University, Taichung 404333, Taiwan; ^15^Department of Pharmacology, China Medical University, Taichung 404333, Taiwan

## Abstract

Pancreatic cancer has higher incidence and mortality rates worldwide. PW06 [(E)-3-(9-ethyl-9H-carbazol-3-yl)-1-(2,5-dimethoxyphenyl) prop-2-en-1-one] is a carbazole derivative containing chalcone moiety which was designed for inhibiting tumorigenesis in human pancreatic cancer. This study is aimed at investigating PW06-induced anticancer effects in human pancreatic cancer MIA PaCa-2 cells *in vitro*. The results showed PW06 potent antiproliferative/cytotoxic activities and induced cell morphological changes in a human pancreatic cancer cell line (MIA PaCa-2), and these effects are concentration-dependent (IC_50_ is 0.43 *μ*M). Annexin V and DAPI staining assays indicated that PW06 induced apoptotic cell death and DNA condensation. Western blotting indicated that PW06 increased the proapoptotic proteins such as Bak and Bad but decreased the antiapoptotic protein such as Bcl-2 and Bcl-xL. Moreover, PW06 increased the active form of caspase-8, caspase-9, and caspase-3, PARP, releasing cytochrome c, AIF, and Endo G from mitochondria in MIA PaCa-2 cells. Confocal laser microscopy assay also confirmed that PW06 increased Bak and decreased Bcl-xL. Also, the cells were pretreated with inhibitors of caspase-3, caspase-8, and caspase-9 and then were treated with PW06, resulting in increased viable cell number compared to PW06 treated only. Furthermore, PW06 showed a potent binding ability with hydrophobic interactions in the core site of the Fas-Fas death domains (FADD). In conclusion, PW06 can potent binding ability to the Fas-FADD which led to antiproliferative, cytotoxic activities, and apoptosis induction accompanied by the caspase-dependent and mitochondria-dependent pathways in human pancreatic cancer MIA PaCa-2 cells.

## 1. Introduction

Pancreatic cancer is one of the highest mortality rates in the cancer population worldwide. Recently, pancreatic ductal adenocarcinoma accounts for 3% of all new cancer cases and is responsible for 8% of cancer deaths in 2020 [1]. In 2018, it accounted for 458,918 new cases of pancreatic cancer worldwide. However, it caused about 432,242 patients to die [2]. Moreover, pancreatic cancer is often diagnosed in patients with terminal stages that led to difficult-to-curable surgery. Thus, it caused higher patient death [3]. However, the overall five-year survival rate is less than 10% [4]. It has been predicted to become the second leading cause of cancer mortality in developed countries [5]. Currently, the curative treatment for pancreatic cancer patients is surgical resection followed by adjuvant chemotherapy [6]. Nevertheless, the cure rate is still unsatisfactory. Thus, it is urgently needed to develop nonsurgical therapeutic approaches to effectively treat patients with pancreatic cancer.

Apoptosis is programmed cell death and is also referred to as a process of cell suicide. Apoptotic proteins involved in cellular demise by which the body maintains the homeostasis of the internal environment are triggered by a range of internal and external factors of cells [7]. Numerous clinical chemotherapeutic anticancer drugs have been known to induce apoptotic death in cancer cells [8]. Currently, apoptosis can be classified into the death receptor pathway, endoplasmic reticulum stress pathway, and mitochondria pathway [9, 10]. The death receptor pathway, also known as an extrinsic pathway, is triggered by agents via death receptors such as Fas-Fas death domains (FADD), TNF (tumor necrosis factor), and TRAILR 1 (tumor necrosis factor-related apoptosis-inducing ligand receptor 1) [11]. Upon Fas and ligand engagement, which activated the intracellular signaling of triggering the initiator caspase-8 then moves downstream for cell apoptosis [12]. The endoplasmic reticulum stress pathway of apoptosis was developed via the IRE1/apoptosis signal-regulating kinase 1 (ASK1)/JNK pathway, the caspase-12 kinase pathway, and the CCAAT/enhancer-binding protein homologous protein (CHOP)/growth arrest- and DNA damage-inducible gene 153 (GADD153) pathway [13, 14]. The mitochondrial pathway, also known as the intrinsic apoptosis pathway, is triggered by DNA damage and activation of p53, followed by a cascade of events leading to mitochondrial outer membrane permeabilization for releasing apoptosis factors [15].

Caspases are intrinsically involved in the mitochondrial pathway. Caspase-9 is the initiative protein in the caspase family, triggering the activation of caspase-3 leading to apoptosis development [9]. B cell leukemia/lymphoma 2 (Bcl-2) family of proteins has involved the regulation of apoptosis through activities of their proapoptotic proteins (Bak and Bax) and antiapoptotic proteins (Bcl-2 and Bcl-xL) by operating independently or dependently between internal death signals and the cell's surface for ensuring a balance [16].

Some carbazole alkaloids have been reported to inhibit the motility, migration, and proliferation of human pancreatic cancer cells [17–19]. Moreover, chalcone possesses anticancer in human pancreatic cancer [20, 21]. PW06 [(E)-3-(9-ethyl-9H-carbazol-3-yl)-1-(2,5-dimethoxyphenyl) prop-2-en-1-one] is a carbazole derivative containing chalcone moiety chemical compound that was first designed and synthesized for inhibiting tumorigenesis in human pancreatic cancer. Therefore, in the present study, we aim to investigate the molecular mechanism of PW06-induced apoptosis effects on human pancreatic carcinoma MIA *PaCa-2* cells. Herein, we also have attempted to define the chemotherapeutic and chemosensitizing potential of PW06 on MIA PaCa-2 cells *in vitro*.

## 2. Materials and Methods

### 2.1. Chemicals and Reagents

**Figure 1:**
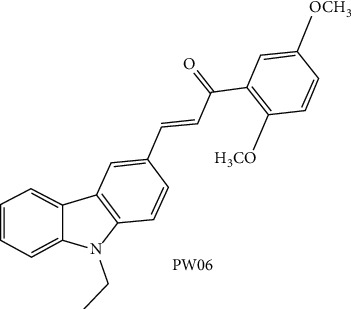
The chemical structure of PW06.

PW06 (Figure 1) was generated by Dr. Jin-Cherng Lien (College of Pharmacy, China Medical University, Taichung, Taiwan). DAPI, propidium iodide (PI), and trypsin-EDTA were purchased from Sigma Chemical Co. (St. Louis, MO, USA). Dulbecco's modified Eagle's medium (DMEM), fetal bovine serum (FBS), L-glutamine, and penicillin-streptomycin were purchased from GIBCO®/Invitrogen Life Technologies (Carlsbad, California, USA). Primary antibodies against -Bid, -Bax, -p53, -Bcl-2, -Bcl-xL,-XIAP, -cytochrome c, -caspase-9, -caspase-3, -caspase-8, -PARP, -Fas, -Endo G, and peroxidase-conjugated secondary antibodies were purchased from Cell Signaling Technology, Inc. (Beverly, MA, USA). Alternatively, primary antibodies against *β*-actin, Bcl-xL, Bak, and AIF were purchased from Sigma-Aldrich (St. Louis, MO, USA).

### 2.2. NMR Spectra of PW06

1H-NMR (CDCl3-d1, 400 MHz) (ppm) *MS (m/z): 385 (M+):* 1.42 (t, J =7.2 Hz, 3H, -CH2CH3), 3.81 (s, 3H, 5'-OCH3), 3.86 (s, 3H, 2'-OCH3), 4.35 (q, J =7.2 Hz, 2H, -CH2CH3), 6.94 (d, J =8.8 Hz, 1H, H-3'), 7.01 (dd, J =8.8, 2.8 Hz, 1H, H-4'), 7.18 (d, J =2.8 Hz, 1H, H-6'), 7.26 (dd, J =7.2, 7.2 Hz, 1H, H-6), 7.36-7.42 (m, 3H, H-1,8, CH=CH-C=O), 7.48 (dd, J =7.6, 7.6 Hz, 1H, H-7), 7.73 (d, J =8.4 Hz, 1H, H-2), 7.83 (d, J =15.6 Hz, 1H, CH=CH-C=O), 8.09 (d, J =7.6 Hz, 1H, H-5), 8.28 (s, 1H, H-4).

### 2.3. Cell Culture

The human pancreatic carcinoma cell line (MIA *PaCa-2*) was obtained from the Food Industry Research and Development Institute (Hsinchu, Taiwan). The cells were cultured in Dulbecco's modified Eagle's medium (DMEM) supplemented with 10% fetal bovine serum (FBS) (HyClone, Logan, UT, USA), 100 *μ*g/ml streptomycin, 100 units/ml penicillin, and 2 mM L-glutamine, incubated at 37°C in a humidified incubator containing 5% CO_2_chamber. The cells were subcultured following the standard protocol.

### 2.4. Cell Morphology and Viability Assays

MIA *PaCa-2* cells (2 × 10^5^ cells/well) were kept and treated in 12-well plates with DMEM medium with PW06 (0, 0.2, 0.4, 0.6, 0.8, and 1.0 *μ*M) for 24 and 48 h. Cell morphological changes were examined and photographed under phase contrast microscopy at 200×. After cells were photographed, the cells from each treatment were collected for measuring the total viable cell number by MTT assay (viable cells reduce 3-(4,5-dimethylthiazol-2-yl)-5-(3-carboxymethoxyphenyl)-2-(4-sulfophenyl)-2H-tetrazolium (MTS) to a formazan product) as previously [22]. Each condition was tested 3 times.

### 2.5. Cell Apoptosis Was Measured by Annexin V Staining

Annexin V-FITC apoptosis detection kit examined apoptotic cell death as described previously [16]. Moreover, staining was performed by the method described in the manufacturer's instructions. In brief, MIA PaCa-2 cells (2 × 10^5^ cells/ml) were plated onto 24-well culture plates for 24 h and were incubated with 0, 0.2, 0.4, and 0.8 *μ*M of PW06 for 48 h. The cells from each treatment were collected and resuspended in annexin V binding buffer and were incubated with annexin V-FITC for 15 min in the dark, and apoptotic cell death was analyzed using BD FACS Calibur as described previously [16]. The apoptosis rate was further analyzed using MultiCycle software (Beckman Coulter, Brea, CA, USA).

### 2.6. DAPI Staining

DAPI staining measured cell nuclear condensation and fragmentation as previously described [23]. In brief, MIA PaCa-2 cells (2 × 10^5^ cells/well) were seeded in the 12-well plates for 24 h and were incubated at 0, 0.2, 0.4, and 0.8 *μ*M of PW06 for 48 h with three replicated wells set in each treatment. The cells were washed with PBS and were fixed in 3% paraformaldehyde in PBS for 20 min at room temperature, washed, collected, spread onto slides, and stained with DAPI solution (2 *μ*g/ml) in the dark for 5 min, and then examined and photographed using a fluorescence microscope.

### 2.7. Western Blotting Analysis

MIA PaCa-2 cells (1 × 10^6^ cells/well) were plated in a 12-well plate for 24 h and were incubated with PW06 (0, 0.2, 0.4, 0.6, and 0.8 *μ*M) for 48 h, and the cells were collected. Isolated cells were lysed, and total protein was quantitated using a Bio-Rad protein assay kit (Hercules, California, USA) described previously [24]. A 30 *μ*g of protein was electrophoresed on 12% (*v*/*v*) SDS-polyacrylamide gel (PAGE) and was then electrotransferred to polyvinylidene difluoride (PVDF) membranes. Membranes were blocked with PBST (PBS, 0.05% Tween-20) containing 5% nonfat dry milk and were probed with primary antibodies such as anti-Bid, -Bak, -Bax, -p53, -Bcl-2, -Bcl-xL, -XIAP, -cytochrome c, -caspase-9, -caspase-3, -caspase-8, -PARP, -Fas, -Endo G, and -AIF or *α*-tubulin in 0.1% PBST (1 : 1000) for 1 h at room temperature. The membranes were washed three times in TBS containing 0.05% Tween 20. At the end of washing, the appropriate peroxidase-labeled conjugated anti-mouse IgG (Santa Cruz Biotechnology) secondary antibody (1 : 1000) was added to the membrane for 1 h at room temperature. Specific bands (antibody-reactive bands) were enhanced using chemiluminescence signal reagents using ECL detection (Amersham Biosciences ECLTM) and using Bio-Rad ChemiDoc™ System for chemiluminescence western blot detection, and densitometry was performed as described previously [24].

### 2.8. Cells Were Pretreated with the Inhibitors of Caspase-3, Caspase-8, and Caspase-9 for Measuring Cell Viability

MTT assay measured the total viable cell number in MIA PaCa-2 cells as described previously [25]. In brief, MIA PaCa cells (2 × 10^5^ cells/well) were kept in a 12-well plate and were pretreated with the inhibitors of caspase-8, caspase-9, and caspase-3 (z-IETD-fmk, z-LEHD-fmk, and z-DEVD-fmk, respectively) and then were incubated with or without PW06 for 48 h, and the cells were assayed the total viable cell number by using MTT assay.

### 2.9. Immunofluorescence Staining

MIA PaCa-2 cells (1.5 × 10^4^ cells/well) were placed on a 6-well plate and treated with 0 and 0.4 *μ*M of PW06 for 48 h; the cells were fixed in 4% formaldehyde in PBS for 15 min and permeabilized for 1 h with 0.3% Triton-X 100 in PBS. After that, the cells were washed and stained with anti-Bcl-xL and -Bak (green fluorescence) followed with FITC-conjugated goat anti-mouse IgG (secondary antibody). The nucleus was stained by DAPI (blue fluorescence) and PI (red fluorescence) for double-check, and Leica TCS SP2 Confocal Spectral Microscope was used to examine photomicrography as described previously [26]. The localization of Bcl-xLor Bak in the cytosol was formed in green color based on blue and red overlapped pixels.

### 2.10. Protein-Ligand Docking Assay

The molecular docking software PyRx version 0.98 for AutoDock Vina was used for all docking calculations [27]. The ligand structure of PW06 was created from the Reaxys database (Elsevier, NL). The receptor structure, FasL (PDB: 3EZQ), was obtained from the RCSB Protein Data Bank [28]. The AutoDock Vina automatically samples different conformations of the ligands to fit the predicted binding site best. The docking results were analyzed and based on the protein-ligand complex's binding affinity (kcal/mol). Docked complexes were visualized and analyzed using the PyMOL Molecular Graphics System (Ver. 2.4 Schrödinger, Portland, OR, USA) [29]. The interactions between protein and ligand were analyzed using the “LIGPLOT” module within the LigPlot+ program (v2.2) [30]. The receptor structure and ligand were prepared, and then, docking was performed into a grid box space with *x*-, *y*, and *z*-axes, and dimensions were adjusted to 73.07 Å × 87.33 Å × 98.34 Å. The docking simulation process with the exhaustiveness parameter is set to 50, and the number of modes is set to 9.

### 2.11. Statistical Analysis

Each of the experiment and assay was done at least three times, and all data were presented as means ± standard deviation (SD). Student's *t*-test carried out statistical analysis, with the following significance levels: ^∗^*p* < 0.05.

## 3. Results

### 3.1. PW06 Induced Cell Morphological Changes and Decreased Cell Viability in MIA PaCa-2 Cells

**Figure 2:**
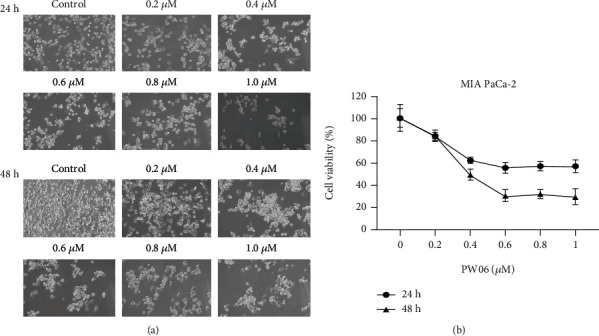
PW06 affected cell morphology and decreased cell viability of MIA PaCa-2 cells. The cells were treated with PW06 (0, 0.2, 0.4, 0.6, 0.8, and 1.0 *μ*M) for 24 and 48 h. Cell morphological changes were examined and photographed under phase contrast microscopy at x400 (a). The cells were collected for measuring the total viable cell number (b) by MTT assay as described in Materials and Methods. The IC_50_ is around 0.43 *μ*M for 48 h.

MIA PaCa-2cells were treated with various concentrations (0, 0.2, 0.4, 0.6, 0.8, and 1.0 *μ*M) for 24 and 48 h. The cells were examined and photographed for morphological changes as shown in Figure 2(a). The total cell viability was further measured, and the results are presented in Figure 2(b). The results showed that PW06 significantly induced cell morphological changes based on reduced cell number, increased cell debris, and developed cells shrunken or aggregated in a concentration-dependent manner presented in Figure 2(a). Moreover, in Figure 2(b) after the calculation of the total viable cell number indicated that PW06 decreased the total viable cell number of MIA PaCa-2 cells around16.4-70.6% for 48 h, these effects are concentration-dependent, and IC_50_ is around 0.43 *μ*M.

### 3.2. PW06 Induces Apoptotic Cell Death in MIA PaCa-2 Cells

**Figure 3:**
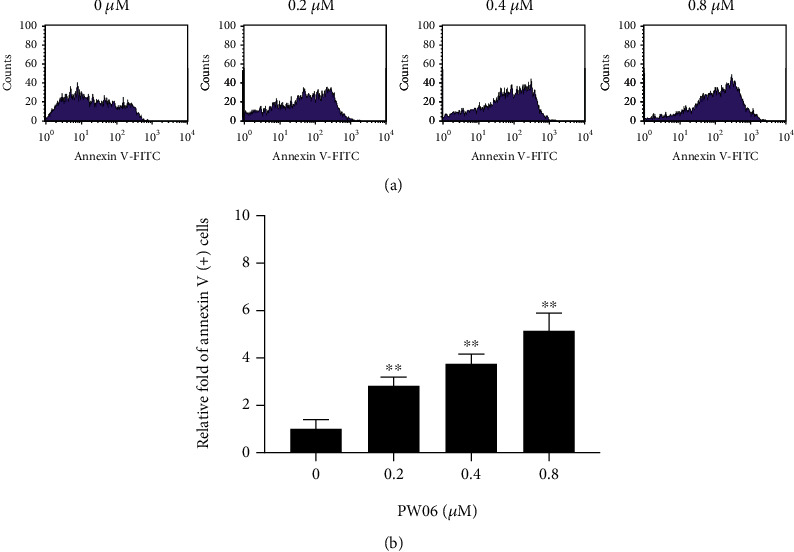
PW06 induces apoptotic cell death in MIA PaCa-2 cells. The cells were treated with PW06 (0, 0.2, 0.4, and 0.8 *μ*M) for 48 h and were measured apoptotic cell death using annexin-V staining as described in Materials and Methods. (a) Profiles of flow cytometric assay. (b) The relative fold of apoptotic cell death. ^∗∗^*p* < 0.01, the significant difference between PW06-treated groups and the control as analyzed by Student's *t*-test.

PW06 reduced total cell viability through apoptotic cell death in MIA PaCa-2 cells for further confirmation. The cells were treated with PW06 (0, 0.2, 0.4, and 0.8 *μ*M) for 48 h and were collected to measure the apoptotic cell death using annexin-V staining and further analyzed by flow cytometry; the results are shown in Figure 3. The data indicated that PW06 treatment leads to an increase in the number of apoptotic cell death from 2.85- to 5.16-folds compared to control groups. These effects are concentration-dependent in MIA PaCa-2 cells.

### 3.3. PW06 Induces the Chromatin Condensation in MIA PaCa-2cells

**Figure 4:**
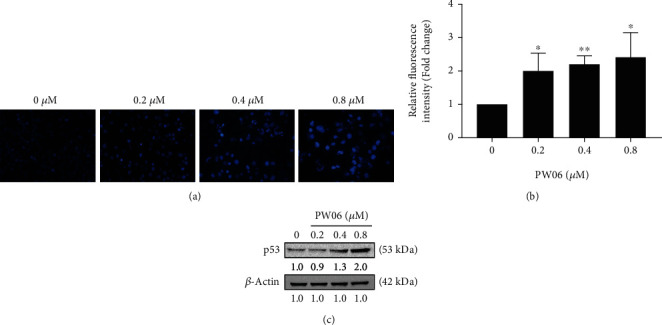
PW06 induces chromatin condensation in MIA PaCa-2 cells. The cells were treated with PW06 (0, 0.2, 0.4, and 0.8 *μ*M) for 48 h and were stained with DAPI. (a) Moreover, photographed using fluorescence microscopy, their intensities of DAPI fluorescence were measured. (b) The relative fluorescence intensity of chromatin condensation. ^∗^*p* < 0.05 and ^∗∗^*p* < 0.01, the significant difference between PW06-treated groups and the control as analyzed by Student's *t*-test. (c) The cells have also measured the expression of p53 as described in Materials and Methods.

MIA PaCa-2 cells were treated with PW06 (0, 0.2, 0.4, and 0.8 *μ*M) for 48 h and were stained with DAPI followed by being examined and photographed using fluorescence microscopy and relative fluorescence as shown in Figure 4(a). And the cells were harvested to investigate p53 expression by western blotting, shown in Figure 4(b). The results indicated that the brighter fluorescence and relative fluorescence of MIA PaCa-2 cells after 48 h of PW06 treatment have higher than that of the control; the effects were from 1.97- to 2.41-folds in a concentration-dependent manner (Figure 4(a)). Bright fluorescence meant the presence of nicked DNA (DNA damage) and chromatin condensation. Thus, we further investigated the expression of p53, and the results indicated that PW06-treated cells have a higher expression of p53 than the control (Figure 4). These observations demonstrated that PW06 reduced total viable cell number via the induction of cell DNA damage, condensation, and apoptosis in MIA PaCA-2 cells.

### 3.4. PW06 Altered the Apoptosis-Associated Protein Expression in MIA PaCa-2 Cells

**Figure 5:**
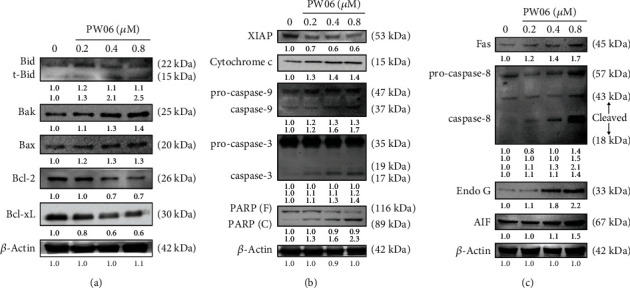
PW06 altered apoptosis-associated protein expression in MIA PaCa-2 cells. The cells were treated with 0, 0.2, 0.4, and 0.8 *μ*M of PW06 for 24 h, and protein expressions were examined by western blotting as described in Materials and Methods. (a) Bid, Bak, Bax, Bcl-2, and Bcl-xL. (b) XIAP, cytochrome c, caspase-9, caspase-3, and PARP. (c). Fas, caspase-8, Endo G, and AIF.

To further confirm whether or not PW06-induced apoptotic cell death is involved in the molecular mechanisms in MIA PaCa-2 cells with apoptosis-associated protein expressions, that was examined by western blotting, and the results are shown in Figures 5(a)–5(c).  Figure 5(a) indicates that PW06 increased the proapoptotic proteins such as tBid, Bak, and Bax. However, it inhibited antiapoptotic proteins such asBcl-2 and Bcl-xL expression in MIA PaCa-2 cells, thus promoting apoptotic. Moreover, PW06 increased cytochrome c, the active form of caspase-9, caspase-3, and PARP, but decreased XIAP (Figure 5(b)), leading to cell apoptosis.  Figure 5(c) indicates that PW06 increased Fas, the active form of caspase-8 that triggers cell apoptosis. Furthermore, it also increased Endo G and AIF for cell apoptosis in MIA PaCa-2 cells (Figure 5(c)). These results indicated that PW06 induced apoptotic cell death in MIA PaCa-2 cells.

### 3.5. PW06 Influenced the Expression of Bcl-xL and Bak in MIA PaCa-2 Cells

**Figure 6:**
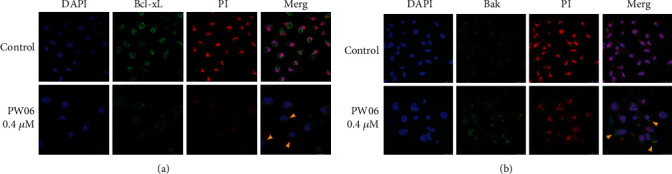
PW06 influenced the expression of Bcl-xL and Bak in MIA PaCa-2 cells. The cells were treated with 0 and 0.4 *μ*M of PW06 for 48 h, and the cells were stained by anti-Bcl-xL (a) and -Bak (b) and then were stained with secondary antibody (FITC-conjugated goat anti-mouse IgG; green fluorescence). All cells were stained by PI (red fluorescence) and DAPI (blue fluorescence) for nucleus double-check examination and were observed under a Leica TCS SP2 Confocal Spectral Microscope as described in Materials and Methods.

MIA PaCa-2 cells were treated with 0.4 *μ*M of PW06 for 48 h. Then, the expression of Bcl-xL and Bak was examined by confocal laser microscopy, and the results are shown in Figures 6(a) and 6(b). The results indicated that PW06 reduced the expression of Bcl-xL (Figure 6(a)) and increased Bak (Figure 6(b)); both figures indicated that both proteins are involved apoptotic cell death in MIA PaCa-2 cells after being exposed to PW06 *in vitro*.

### 3.6. Pretreatment of Inhibitors of Caspases Led to Increasing Viable Cells in PW06 Treatment of MIA PaCa-2 Cells

**Figure 7:**
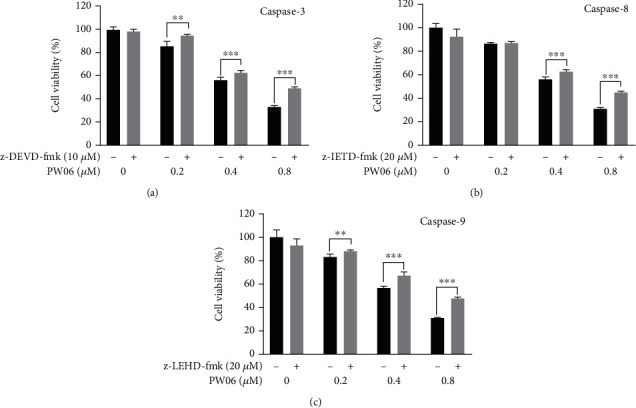
Inhibitors of caspase-3, caspase-8, and caspase-9 affect PW06 and decreased cell viability in MIA PaCa-2 cells. The cells were pretreated with z-DEVD-fmk, z-IETD-fmk, and z-LEHD-fmk and treated with PW06 (0, 0.2, 0.4, and 0.8 *μ*M) for 48 h and were harvested for measuring the cell viability ((a) caspase-3, (b) caspase-9, and (c) caspase-8) using MTT assays as described in Materials and Methods. ^∗∗^*p* < 0.01 and ^∗∗∗^*p* < 0.001, significant difference between pretreated inhibitor groups and the control as analyzed by Student's *t*-test.

To further confirm whether the caspases were involved or not in PW06-induced cell death, MIA PaCa-2 cells were pretreated with inhibitors of caspase-3, caspase-8, and caspase-9. They then were treated with PW06 0, 0.2, 0.4, and 0.8 *μ*M for 48 h, and the cells were collected for measuring the percentage of viable cells. The results are shown in Figures 7(a)–7(c), which indicated that those inhibitors were pretreated, followed by PW06 treatment which increased the viable cells compared to PW06 treatment only. These results also showed that PW06-induced apoptotic cell death might involve the activations of caspase-3, caspase-8, and caspase-9 in MIA PaCa-2 cells.

### 3.7. Effects of PW06 on Fas-FADD with Protein-Ligand Docking

**Figure 8:**
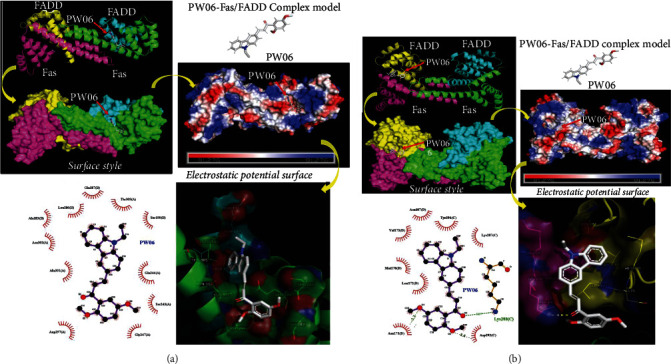
Effects of PW06 on Fas-FADD with protein-ligand docking. There are two types of receptor docking situations of PW06 binding models to Fas-FADD models. (a) and (b) The protein-ligand docking models indicated that PW06 has 2 binding situations models to the tetrameric arrangement of Fas-FADD. The results come through PW06 at least has 6 binding models to tetrameric arrangement of Fas-FADD in two major binding situations, respectively. The binding site was established as a surface model, and the inhibitor was displayed as a ball and stick model. The green-dashed line indicates the hydrogen bond pairing with each other. The red circles identify the residues on each plot that was equivalent. Red or pink eyebrow-like icons illustrate hydrophobic interactions. The details of the experimental preform were described in Materials and Methods.

To further characterize how PW06 interacts with Fas-FADD, molecular docking analysis was conducted to evaluate potential interaction sites with PyRx. The program proposed two potential models which are shown in Figure 8. Model 1 predicts that the carbazole end of the PW06 may be inserted into the cavity between Fas and FADD and interacts with Ala183, Leu186, Gln187, and Ser190 in FADD and Asn302 and Thr305 in FAS through hydrophobic interactions (Figure 8(a)). Besides, PW06 also has hydrophobic interactions with Ser243, Gln244, Gly247, Asp297, and Ala301 in FAS. Simulation results indicated that the best binding affinity of PW06 was -7.3 kcal/mol. On the other hand, model 2 predicts that PW06 may be inserted into the groove between Fas and FADD and interacts with Asn107, Met170, Asn171, Leu172, and Val173 in FADD and Lys287, Lys288, Tyr291, and Asp292 in FAS through hydrophobic interactions and forms a hydrogen bond with Lys288 (Figure 8(b)). In this model, the best binding affinity of PW06 was -6.7 kcal/mol.

## 4. Discussion

Currently, many clinical drugs are obtained from natural products. Moreover, numerous studies also showed that dietary fruits and vegetables, including flavonoids, may be used as potential candidates for developing chemotherapeutic agents [31]. However, many current clinical drugs still induced side effects; thus, selecting clinically used drugs for further synthesis to reduce side effects will increase the efficacy for patients. Therefore, herein, the present study set the synthesized compound (PW06) for investigating whether or not it can induce cell death; thus, the first experiments showed that PW06 decrease in viability observed in the cell line (MIA PaCa-2) could be due to a reduction in cell proliferation or an increase in cell death and senescence; we further analyzed the effect of PW06 on cell apoptosis and further examining associated protein expression in MIA PaCa-2 cells.

In the present study, we found that (1) PW06 induced cell morphological change and decreased total cell viability (Figures 2(a) and 2(b)); (2) induced apoptotic cell death was examined by annexin V staining (Figure 3); (3) induced DNA condensation (Figure 4(a)) and increased p53 (Figure 4(b)); (4) induced proapoptotic proteins expressions such as Bak and Bax but decreased antiapoptotic proteins such as Bcl-2 and Bcl-xL (Figure 5(a)); increased active form of caspase-8, caspase-9, and caspase-3 (Figure 5(b)); and increased Fas, the active form of Endo G, AIF, and PARP (Figure 5(c)); (5) confocal laser microscopy examining indicated that PW06 decreased Bcl-xL and increased Bak (Figures 6(a) and 6(b)) in MIA PaCa-2 cells; and (6) the cells were pretreated with inhibitors of caspase-3, caspase-8, and caspase-9 and treated with PW06 leading to increase viable cells (Figures 7(a)–7 (c)).

The effects of PW06 on MIA PaCa-2 cells were examined through the changes of viable cells and detected the morphological cell charges.  Figure 2 indicates that PW06 significantly induced cell morphological changes based on the examined and photographed under phase contrast microscopy. To further investigate, PW06 decreased total cell viability. Thus, annexin V and DAPI staining assays were performed to investigate apoptotic cell death and DNA condensation, respectively, and the results are presented in Figures 3 and 4. Annexin V assay for cell apoptosis and DAPI staining are accepted protocols for investigating cell apoptosis based on the chromatin condensation [32, 33]. Figures 4(a) and 4(b) indicate that PW06 induced chromatin condensation (apoptotic cells) in MIA PaCa-2 cells.

It is well documented that various antiapoptotic (Bcl-2, Bcl-xL, Mcl-1, etc.) and proapoptotic (Bax, Bak, Bim, Bid, etc.) proteins regulate intrinsic/mitochondrial pathways [34, 35]. Therefore, one of the pathways associated with apoptosis factors attended to be targeted for cancer therapy is the anti-apoptotic B-cell lymphoma-2 (Bcl-2) family of proteins, including Bcl-w and Bcl-xL Bfl1/A-1, Bcl-2, Mcl-1, and Bcl-B [36]. Bax is a pore-forming and mitochondria-associated protein, a death-promoting member named proapoptotic protein [37]. Mitochondrial dynamics have also been associated with cell death. Bcl-xL, an antiapoptotic protein, was found on the mitochondrial membrane, and it prevents mitochondria-dependent death processes by blocking the oligomerization of prodeath proteins, including Bax and Bak [38, 39]. Based on pieces of literature, they have shown a connection between Bcl-2 family members (Bax, Bak, Bcl-2, and Bcl-xL) and proteins involved in mitochondrial morphogenesis [40, 41]. In this aspect, PW06 might cause cell death through the changes of ratio Bax/Bcl-2 and caspase activation, followed by cell apoptosis in MIA PaCa-2 cells. Therefore, PW06-induced cell apoptosis also involved the Bcl-2 family in MIA PaCa-2 cells *in vitro* (Figure 5(a)). Furthermore, Figure 5(a) also shows that PW06 increased the proapoptotic proteins (tBid, Bak, and Bax) but inhibited antiapoptotic protein (Bcl-2 and Bcl-xL) expression in MIA PaCa-2 cells. Moreover, the confocal laser microscopy system also confirmed that PW06 increased Bak and decreased Bcl-xL in MIA PaCa-2 cells (Figure 6).

Furthermore, the overexpression of Bcl-2 and Bcl-xL will suppress the proapoptotic mitochondria. It could result in cytosolic accumulation of cytochrome c. Moreover, the release of cytochrome c will open the mitochondrial permeability transition pore [42]. Bax has been shown to trigger cytochrome c from isolated mitochondria [43]. The agents reduced the Bax/Bcl-2 ratio but increased the level of XIAP and cleaved caspase-3/caspase-9; and cleaved PARP can induce apoptosis [44]. For further investigation which were the cell apoptosis-associated proteins involved in PW06-induced cell apoptosis in MIA PaCa-2 cells. Thus, western blotting was used to investigate the protein expression, increasing Fas and the active form of caspase-8, caspase-9, and caspase-3 in MIA PaCa-2 cells after exposure to PW06 for 48 h, and the results are presented in Figure 5(b). Therefore, we suggested that PW06 induced apoptotic cell death by activating caspase dependently.

In mitochondria, cytochrome c, Endo G, and AIF were followed by the activation of caspases-9 and caspase-3, and the apoptotic cell death was developed which was named intrinsic pathway apoptosis [45]. Z-VAD-fmk is a pancaspase inhibitor [46], z-IETD-fmk is a caspase-8 inhibitor, and Z-LEHD-fmk is a caspase-9 inhibitor [47] that prevents apoptosis in many different cell types. For further exploration which were the mitochondria-associated proteins were related to PW06-induced cell apoptosis in MIA PaCa-2 cells. Thus, PW06 increased Fas, cytochrome c, Endo G, AIF, and cleaved form of PARP but decreased the XIAP expression in MIA PaCa-2 cells, and the results are presented in Figure 5(c). Thus, Fas are involved in PW06-induced apoptosis *via* the extrinsic pathway of apoptosis.

Activated Fas and FADD can promote procaspase-8 transfer to caspase-8 and activate two apoptotic pathways, the extrinsic and the intrinsic pathways [48]. The Fas-FADD complex consists of two tetrameric assemblies containing four Fas death domains and four FADD death domains in these two asymmetric assembly units [49]. The global docking results showed that PW06 is mainly bound in the gap between the Fas-FADD interface. In two potential binding models, PW06 can form a stable complex with Fas-FADD via hydrophobic interaction and hydrogen bonding (Figures 8(a) and 8(b)). And these simulation results can be combined with the above experimental data to illustrate the binding ability of PW06 to Fas-FADD.

**Figure 9:**
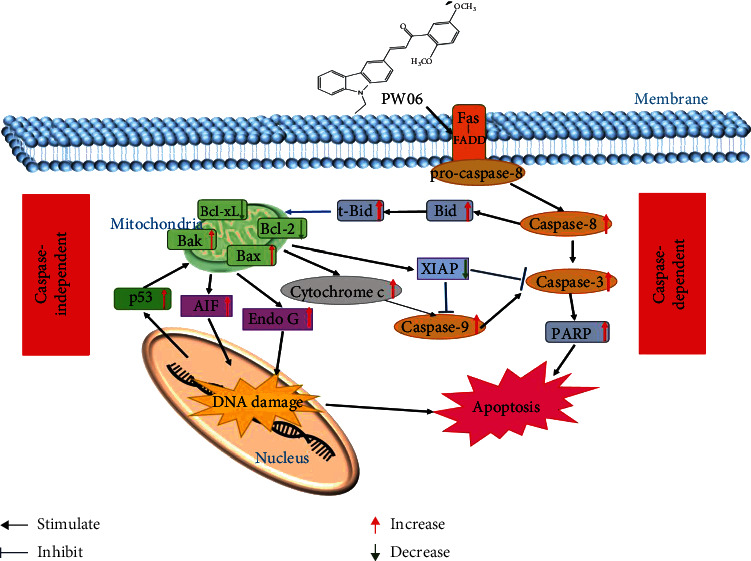
The possible signaling pathways for PW06-induced apoptotic cell death in MIA PaCa-2 cells *in vitro*.

Thus, the apoptosis pathway assumes that PW06 interacted with the Fas-FADD proteins and then activates the caspase-independent apoptotic pathway (mitochondria-related pathway) and the caspase-dependent apoptotic pathway in human pancreatic cancer MIA PaCa-2 cells. Overall, the possible apoptosis signaling pathways of PW06 on MIA PaCa-2 cells are summarized in Figure 9.

## Data Availability

The data used to support the findings of this study are included within the article.
